# Consensus on early detection of disease progression in patients with multiple sclerosis

**DOI:** 10.3389/fneur.2022.931014

**Published:** 2022-07-28

**Authors:** José E. Meca-Lallana, Bonaventura Casanova, Alfredo Rodríguez-Antigüedad, Sara Eichau, Guillermo Izquierdo, Carmen Durán, Jordi Río, Miguel Ángel Hernández, Carmen Calles, José M. Prieto-González, José Ramón Ara, Dionisio F. Uría, Lucienne Costa-Frossard, Antonio García-Merino, Celia Oreja-Guevara

**Affiliations:** ^1^CSUR Multiple Sclerosis and Clinical Neuroimmunology Unit, Neurology Department, Hospital Clínico Universitario Virgen de la Arrixaca, IMIB-Arrixaca, Murcia, Spain; ^2^Department of Neurology, Hospital Universitario y Politécnico La Fe, Valencia, Spain; ^3^Department of Neurology, Hospital Universitario Cruces, Barakaldo, Spain; ^4^Department of Neurology, Hospital Universitario Virgen Macarena, Sevilla, Spain; ^5^Fundación DINAC, Sevilla, Spain; ^6^Department of Neurology, Hospital Universitario de Badajoz, Badajoz, Spain; ^7^CEMCAT, Hospital Universitario Vall d'Hebrón, Barcelona, Spain; ^8^Department of Neurology, Hospital Universitario Nuestra Señora de la Candelaria, Santa Cruz de Tenerife, Spain; ^9^Department of Neurology, Hospital Universitario Son Espases, Palma de Mallorca, Spain; ^10^Department of Neurology, Hospital Clínico Universitario de Santiago, Santiago de Compostela, Spain; ^11^Department of Neurology, Hospital Universitario Miguel Servet, Zaragoza, Spain; ^12^Department of Neurology, Hospital Universitario de Cabueñes, Gijón, Spain; ^13^Department of Neurology, Hospital Universitario Ramón y Cajal, Madrid, Spain; ^14^Department of Neurology, Hospital Universitario Puerta de Hierro, Madrid, Spain; ^15^Department of Neurology, Hospital Universitario Clínico San Carlos, Madrid, Spain

**Keywords:** multiple sclerosis, early detection, secondary progressive multiple sclerosis, consensus, disease progression

## Abstract

**Background:**

Early identification of the transition from relapsing-remitting multiple sclerosis (RRMS) to secondary progressive MS (SPMS) can be challenging for clinicians, as diagnostic criteria for SPMS are primarily based on physical disability and a holistic interpretation.

**Objective:**

To establish a consensus on patient monitoring to identify promptly disease progression and the most useful clinical and paraclinical variables for early identification of disease progression in MS.

**Methods:**

A RAND/UCLA Appropriateness Method was used to establish the level of agreement among a panel of 15 medical experts in MS. Eighty-three items were circulated to the experts for confidential rating of the grade of agreement and recommendation. Consensus was defined when ≥66% agreement or disagreement was achieved.

**Results:**

Consensus was reached in 72 out of 83 items (86.7%). The items addressed frequency of follow-up visits, definition of progression, identification of clinical, cognitive, and radiological assessments as variables of suspected or confirmed SPMS diagnosis, the need for more accurate assessment tools, and the use of promising molecular and imaging biomarkers to predict disease progression and/or diagnose SPMS.

**Conclusion:**

Consensus achieved on these topics could guide neurologists to identify earlier disease progression and to plan targeted clinical and therapeutic interventions during the earliest stages of SPMS.

## Introduction

MS is a chronic, inflammatory, immune-mediated disease of the CNS characterized by demyelination and axonal degeneration (1). Most patients (~85%) initiate with a relapsing–remitting course (RRMS) which can evolve to a secondary progressive form characterized by irreversible disability accumulation independent of relapses (SPMS) (2). Time from disease onset until conversion to SPMS varies widely among studies (3–5). A median time of 32.4 years has been recently reported (3), which is considerably higher than that observed a decade ago (21.4 years) (4), most likely due to the use of more efficacious emerging RRMS treatments.

Identifying the transition from RRMS to SPMS remains a challenge for physicians, as both phenotypes overlap as a continuum, and combined signs of early progression may present differently among patients. Diagnosis is often guided by a confirmed increase in physical disability independently of relapses, decline in cognitive functions, and the onset of persistent symptoms reported by patients. SPMS is thus frequently diagnosed retrospectively, with an estimated average 2–3-year delay between detection of the first signs of suspected progression and confirmed diagnosis of SPMS (6, 7). Several promising cerebrospinal fluid and blood plasma biomarkers have shown great potential as early markers of neurodegeneration and progression independent of relapses and are being integrated as part of the long-term patient monitoring in some specialized MS units (8).

An unequivocal definition of SPMS based on the Expanded Disability Status Scale (EDSS) and previous relapses has been proposed by Lorscheider et al. as a potential tool for timely SPMS diagnosis (7). Despite its accuracy for identifying the onset of progression (87%), the definition relies on the EDSS as a single diagnostic tool, an approach that is not free from limitations (7). Besides the EDSS, other disability-related measures, such as the Timed 25-Feet Walk Test (T25FWT) or 9-Hole Peg Test (9-HPT) significantly predicted conversion to SPMS (9, 10).

The growing knowledge of the underlying pathogenic processes involved in MS progression has led to the development of new drugs targeting SPMS patients (11). To maximize the potential therapeutic impact of such drugs, there is an imperative need to identify and treat SPMS patients in a timely manner. In response to this unmet need, an effort to develop a consensus document by a panel of 15 Spanish MS experts was undertaken.

The main purpose of this consensus is to identify early disease progression to help clinicians in detecting early signs of progression and make the most appropriate and timely therapeutic decisions in their practice. We present here the main topics of agreement on the most relevant aspects for early detection of progression identified by the panel of experts.

## Materials and methods

### Overview of the method of consensus

The RAND/UCLA Appropriateness Method (RAM) was used (12). The RAM is based on the Delphi method and integrates the review of scientific evidence with the opinion of experts regarding the appropriateness of a medical decision and/or intervention. The RAM has previously been applied to formalize the grade of agreement among experts on the management and diagnosis of MS patients (13, 14), and in other diseases (15, 16).

### Expert panel composition

The experts were selected based on their publication record and long-term experience in specialized MS units. The panel was defined to represent the breadth of knowledge, experience, and opinions of national MS experts, covering all national territories.

The working group was divided into two subgroups: a steering committee and a rating group. The former was constituted by 3 experts who were involved in drafting the initial proposal of statements. The latter was formed by 15 experts, including the 3 members of the steering committee, and rated the pre-defined statements (henceforth the experts). The RAND/UCLA method was conducted with an experienced facilitator.

### First stage: Statements definition

The steering committee drafted a list of guidance statements including the identification of clinical features [functional and EDSS assessments [37 statements], cognitive assessments (16), additional assessments (9)], radiological characteristics (7), and biomarkers (7). An on-site meeting of the steering committee was held (25^th^ April, 2019) to share the individual proposals and prepare the first draft of the questionnaire. After the meeting, the proposed statements were reviewed individually by the three members, resulting in the validation of an initial questionnaire with 72 guidance statements.

### Second stage: Statements rating

**Figure 1:**
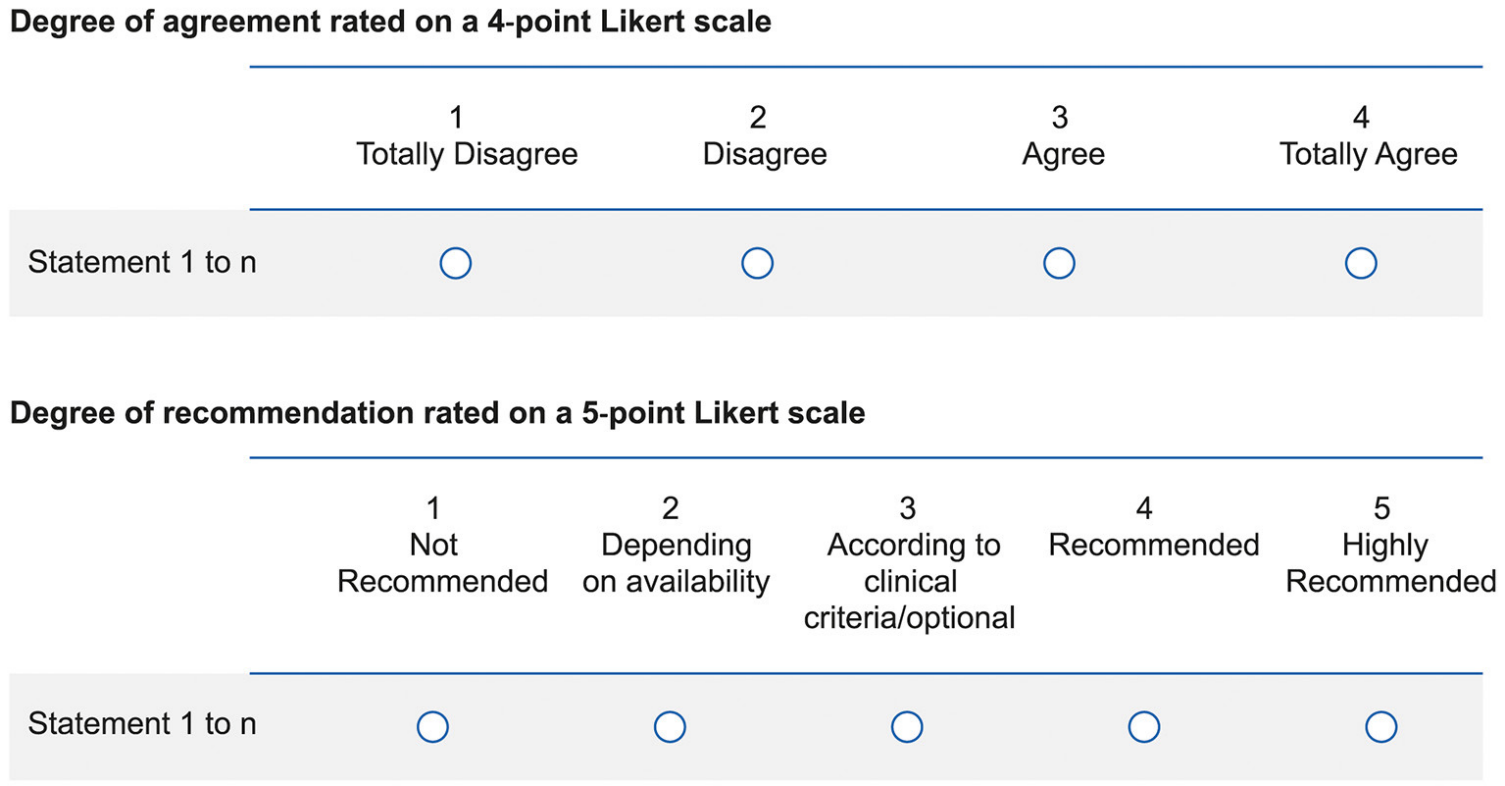
Likert scales used to rate the statements.

The rating group gave feedback on each statement in a two-round process. In the first round, each statement was submitted to the rating group, who privately rated their grade of agreement on a 4-point Likert scale and the grade of recommendation using a 5-point Likert scale (Figure 1). Each member sent the ratings to a facilitator, who integrated the responses that were given in the on-site meeting (16^th^ May, 2019). During this meeting, the rating group discussed their rating, re-rate scores, modify the original list and include new statements; a new version of the questionnaire with 83 statements was created.

In the second round, the revised version of the questionnaire was sent to the experts again, who privately re-rated all the statements and send them to the facilitator. The expert panel was convened for a last on-site meeting, where the results of the ratings for each statement from all the members were shared, the wording of the statements was refined, and the final document with the guidance statements that reached consensus was approved.

A descriptive analysis was conducted. The median value of each statement was calculated based on the numerical value of the 4 or 5 possible ratings in the 4 or 5-point Likert scale, respectively. Based on the median value, statements with a higher proportion of agreement (“Totally agree” and “Agree”) were grouped vs. those with low agreement (“Totally disagree” and “In disagreement”). Consensus in favor was established when the sum of “Totally agree” and “Agree” was ≥66.6% of experts' responses. Consensus against was established when the sum of “Totally disagree” and “In disagreement” was ≥66.6% of experts' responses. A lack of consensus was considered when none of the above assumptions were met.

Likewise, statements with a higher proportion of recommendation (“Recommended” and “Essential”) were grouped vs. those with low recommendation (“Not recommended” and “Depending on availability”). The response “According to clinical criteria/optional” was established as neutral. Consensus in favor was established when the sum of “Recommended” and “Essential” was ≥66.6% of experts' responses, and consensus against when the sum of “Not recommended” and “Depending on availability” was ≥66.6% of experts' responses. A lack of consensus was considered when none of the above assumptions were met. Percentages have been rounded off to whole figures.

## Results

Consensus (grade of agreement) was reached in 72 out of 83 statements (86.7%). Tables 1–3 present the variables in which an isolated change: (i) allows to suspect progression (Table 1), (ii) does not allow diagnosis of progression (Table 2), and (iii) suggests that additional assessments to diagnose progression is required (Table 3). All the statements that reached consensus are shown in Supplementary Tables S1–S7. Also, the statements that did not reach consensus are shown in Supplementary Table S8. The description in this section focuses on summarizing the percentage of experts who agreed with the statements.

**Table 1:** Variables whose isolated change allows to suspect diagnosis of progression.

**Statement**	**Consensus in favor**			
	**Grade of agreement**		**Grade of recommendation**	
	**(%)** ^a^	**Median**	**(%)** ^b^	**Median**
A confirmed worsening of 2 points in any functional system (except the visual system)	80	Agree	87	Recommended
A confirmed worsening of 2 points in any functional system (except the visual system),				
with a disease duration <10 years	93	Agree	NC	Recommended
with a disease duration between 10 and 20 years	87	Agree	73	Recommended
with a disease duration > 20 years	73	Agree	80	Recommended
if the patient is <35 years old	87	Agree	73	Recommended
if the patient is between 35 and 45 years old	87	Agree	80	Recommended
if the patient is > 45 years old	87	Agree	80	Recommended
A confirmed 20% time increase in:				
the 25FTW	93	Agree	80	Recommended
the 9HPT	87	Agree	67	Recommended
the 25FTW and the 9HPT	100	Agree	87	Recommended
the 2MWT	87	Agree	80	Recommended
A confirmed 20% reduction in the SDMT	93	Agree	67	Recommended
A confirmed 20% worsening in at least two subtests of the BRB-N or BICAMS battery	87	Agree	80	Recommended
An isolated worsening of cognitive function	87	Agree	67	Recommended
A change in the degree of brain atrophy that is maintained and/or confirmed over time	80	Agree	71	Recommended
A change in the degree of spinal cord atrophy that is maintained and/or confirmed over time	100	Agree	87	Recommended
The presence of diffuse hyperintensity in the brain white matter or confluence of lesions	80	Agree	NC	Recommended
The presence of meningeal ectopic lymphoid follicles	67	Agree	NC	Recommended

a Sum of the percentages of responses obtained for “Totally agree” and “Agree.” If no consensus was reached (i.e., <66%) NC is shown.

b Sum of the percentages of responses obtained for “Recommended” and “Essential.” If no consensus was reached (i.e., <66%) NC is shown.

**Table 2:** Variables whose isolated change does not allow diagnosis of progression.

**Statement**	**Consensus in favor**			
	**Grade of agreement**		**Grade of recommendation**	
	**(%)** ^a^	**Median**	**(%)** ^b^	**Median**
A confirmed worsening by 2 points in any functional system (except the visual system)	67	Disagree	NC	According to clinical criteria/optional
A confirmed worsening by 2 points in any functional system (except the visual system) with a disease duration <10 years	67	Disagree	NC	According to clinical criteria/optional
A confirmed 20% time increase in:				
the 25FTW	93	Disagree	93	Not recommended
the 9HPT	100	Disagree	NC	Not recommended
the 2MWT	93	Disagree	66	Not recommended
Experiencing repeated falls	93	Disagree	NC	Not recommended
A confirmed 20% reduction in the SDMT	93	Agree	67	Recommended
A confirmed 20% worsening in at least two subtests of the BRB-N or BICAMS battery	87	Agree	80	Recommended

a Sum of the percentages of responses obtained for “Totally disagree” and “Disagree.” If no consensus was reached (i.e., <66%) NC is shown.

b Sum of the percentages of responses obtained for “Not recommended” and “Depending on availability.” If no consensus was reached (i.e., <66%) NC is shown.

**Table 3:** Variables whose isolated change indicates that more accurate progression diagnostic tools should be used.

**Statement**	**Consensus in favor**			
	**Grade of agreement**		**Grade of recommendation**	
	**(%)** ^a^	**Median**	**(%)** ^b^	**Median**
A confirmed reduction from 500 to 300 meters in a patient capable of wandering 500 meters or more without help or rest	100	Totally agree	93	Essential
Transition from walking independently to needing any kind of support or help to walk	100	Totally agree	100	Essential
Changes in the QoL questionnaires	80	Agree	73	Recommended
A worsening of spasticity	87	Agree	73	Recommended
A change in the degree of brain atrophy	93	Totally agree	93	Recommended
A change in the degree of spinal atrophy	93	Totally agree	93	Recommended

a Sum of the percentages of responses obtained for “Totally agree” and “Agree.” If no consensus was reached (i.e., <66%) NC is shown.

b Sum of the percentages of responses obtained for “Recommended” and “Essential.” If no consensus was reached (i.e., <66%) NC is shown.

### Identification of progression by clinical features

#### Functional and EDSS assessments

Experts agreed on monitoring patients who are clinically and radiologically stable when treated with immunomodulator (93%) or immunosuppressant (73%) drugs every 6 months. In those patients with clinical and radiological instability related to the disease-modifying treatment (DMT) or with suspected disease progression, it was recommended to increase monitoring frequency to every 3 months (80%). A consensus was also reached on determining the frequency of these patients' follow-up on a case-by-case basis (>80%).

The EDSS score was considered the best variable to define progression by 93% of the experts and all agreed that based on Lorscheider et al. (7) progression could be defined as an increase in EDSS, by 1 or 0.5 points if the baseline EDSS was ≤ 5.5 or ≥ 6, respectively, considering a minimal EDSS of 4, a minimal pyramidal function of 2 and a confirmation of progression over at least 3 months. However, and regardless of the variable used for the assessment, experts agreed that the minimum time to establish the diagnosis of confirmed disability progression not associated with relapses is 6 months (87%). They also considered that a confirmed worsening of 2 points in any isolated functional system (except the visual system), even without changes in the EDSS, suggests progression (80%), regardless of disease duration [ <10 years [93%], between 10–20 years [87%], > 20 years [73%]] and patient age [<35 years, between 35 and 45 years, > 45 years [87%]]. A confirmed minimum 20% increase in the performance of tests evaluating function (25FTW, 9HPT, or 2-min walk test) considered individually was rated sufficient to suspect progression (>87%) but not to confirm it (>93%). Similarly, experts agreed that if a patient experiences repeated falls, even if the EDSS or other scales remain unchanged, progression of disability should be suspected (100%) but not confirmed (93%). Nonetheless, when some of these variables are considered together, and a confirmed 20% increase in the 25FTW and 9HPT is accompanied by an increase in the EDSS (based on the definition described above), a diagnosis of progression can be confirmed (87%).

#### Cognitive assessments

Experts agreed (80%) on performing at least one annual cognitive assessment that includes the largest number of domains, such as the brief repeatable battery of neuropsychological tests (BRB-N, 93%). If applying the BRB-N is not possible, a shorter neuropsychological battery such as the brief international cognitive assessment for MS (BICAMS, 93%), or the symbol digit modalities tests (SDMT) is recommended (100%). Disease progression can be suspected by a confirmed minimum worsening of 20% in two subtests of the BRB-N or BICAMS batteries (87%), or in the SDMT (93%), but diagnosis based only on results of these tests is not recommended.

#### Other assessments

Experts agreed to evaluate, at least once per year, QoL (80%), depression (73%), fatigue (73%), and spasticity (74%), the latter in case of alterations in the pyramidal functional system. A full consensus was achieved on asking patients proactively and in a structured manner if they have perceived changes in their symptoms that may lead to suspect progression. Seventy-four percent of the experts agreed that changes in fatigue and depression scales rarely confirm the diagnosis of progression.

### Identification of progression by radiological characteristics

A high grade of agreement was reached on suspecting disease progression based on a change in the increase of brain atrophy or spinal cord atrophy. Moreover, experts considered that detecting a change in brain or spinal cord atrophy should indicate that more accurate clinical diagnostic tools of disease progression should be used.

### Identification of progression by biomarkers

Presence of ectopic meningeal lymphoid follicles, serum light-chain neurofilaments (sNfL) levels, and optical coherence tomography (OCT) measurements were rated as valid biomarkers supporting detection or suspicion of progression (73, 87, and 67%, respectively). All experts agreed that data collected from wearables and digital devices will become relevant for early identification of disease progression in the future.

## Discussion

Due to the absence of standard criteria for transition identification from RRMS to a secondary progressive course, the diagnosis of SPMS is retrospective and based entirely on clinical judgment. As the reluctance to diagnose SPMS decreases with the arrival of new treatments specific for SPMS patients, consensus statements on SPMS diagnosis will be a key resource for clinicians on the complex decision-making process during this transition from RRMS to SPMS. Here, a formal consensus method was used to make feasible recommendations for a timely and more accurate identification of disease progression. The expert panel reached consensus on most of the statements and with low variation between the grade of agreement and the grade of recommendation, reflecting the robustness of statement identification.

Statements concerned relevant dimensions such as clinical, radiological and biomarkers. Experts agreed on monitoring patients every 6 months when they are clinically and radiologically stable, and to increase the frequency to every 3 months when patients are unstable or with suspected progression. These follow-ups imply a higher frequency compared to the minimum annual monitoring previously suggested (2). Nevertheless, adaptation of monitoring on a case-by-case basis was also acknowledged, indicating that the frequency should be dictated by the patient's characteristics (17).

In terms of defining SPMS, full consensus was reached on adopting the definition developed by Lorscheider et al. (7) for EDSS ≥ 4, which has proved to enable the diagnosis of SPMS more than 3 years earlier than the diagnosis date assigned by the physician. In Lorscheider et al., (7) reducing the time needed to confirm progression from 6 to 3 months only led to a marginal increase in sensitivity (from 88 to 89%), while decreasing specificity (92 to 86%). Based on their daily clinical practice and healthcare experience, the consensus group agreed that a higher specificity should prevail and thus 6 months was defined as the time needed to establish progression.

Using this definition, a study conducted in 15,717 patients from the MSBase registry showed that older age and longer disease duration, among other factors, were independently associated with an increased risk of SPMS (3). In line with these findings, we agreed that older age or longer disease duration together with a worsening of 2 points in any functional system—excluding the visual system—leads to suspect progression but does not allow to confirm diagnosis (18).

Indeed, no single functional assessment was considered sufficient to diagnose progression. Experts agreed that diagnosis can be confirmed when there is a minimum 20% increase in the 25FTW and the 9HPT, along with an increase in EDSS based on the definition given by Lorscheider et al. (7). This consensus concurs with previous research demonstrating that composite measures of disability progression such as the EDSS-Plus (EDSS, 9HPT and T25FW) refine the identification of disability progression in clinically definite SPMS patients (10). However, no evidence has been generated yet on the superiority of the EDSS-Plus vs. the EDSS alone to measure disability worsening in the RRMS course. The utility of using these measures in the early identification of progression proposed here should be confirmed by future research. The use of composite endpoints is essential in the clinical setting but it also needs to be considered in the design of clinical trials (19). The T25FW and 9HPT are especially suitable to assess disease progression as they do not have practice effects, which allows to assume that changes in scores are due to the patient's status rather than measurement variability (20). Regardless of the variable used, the minimum time to establish the diagnosis of confirmed progression of disability not associated with relapses was agreed to be 6 months.

The evaluation of cognitive functions, such as information processing speed (IPS) by the SDMT, together with the EDSS, probably detects more progression events as they measure different aspects of disability (21). IPS is the main cognitive domain affected by progression in MS (22) and SDMT is one of the most valid and efficient tools to detect its impairment (23). The assumption of an additive value by combining these measurements has been further supported by the absence of a strong correlation between the SDMT and EDSS (20) and by worsening on the SDMT independently from worsening on the EDSS (23). In line with this, we believe that, in addition to functional assessment, cognitive domains should be assessed at least annually in RRMS patients by a neuropsychologist or other trained healthcare professional. The assessment should include as many domains as possible, using batteries such as the BRB-N or BICAMS, and if these batteries cannot be applied due to constraints in time and/or resources, full consensus was reached on applying at least the SDMT. However, all experts agreed on conducting a comprehensive neuropsychological study by a neuropsychologist when progression of cognitive decline is suspected. These statements concur with the recommendations by the National MS Society, which indicate using the SDMT to evaluate progression of cognitive impairment, and performing a more comprehensive assessment when significant cognitive decline is detected (24).

Fatigue, QoL, depression, and spasticity were recommended by experts to be assessed at least annually, even if changes in these measurements do not allow to diagnose progression *per se*. Detection of changes in patient-reported outcomes (PROs) may be useful to predict patients at a higher risk to progress in the near future (25). Asking patients' perception of the progression of their own disability was considered of key importance by all experts. Information from the patients' perspective and their awareness of change could contribute to the early detection of progression onset, and a systematic review of changes in patients' narrative may reveal non-obvious early signs of progression.

At present, brain and spinal cord volume measures have a limited role in MS diagnostic criteria (26) or disease course classification (2). Despite increasing studies showing promising results for the use of MRI markers to detect conversion to SPMS (27, 28), translating group-based results to the individual level is not straightforward (29). Individual cut-off values for brain and spinal cord volume changes discriminating RRMS from SPMS are not yet clearly defined, which hampers their practical application in the clinical setting. However, because global brain volume and cervical cord area are associated with and predict disability, their measurement in clinical practice have been recommended (30). Accordingly, we emphasized the relevance but also the limitations of radiological assessments by considering that detecting changes in brain or spinal cord atrophy and the presence of diffuse hyperintensity or meningeal ectopic lymphoid follicles allow suspicion—but not diagnosis—of disease progression.

Experts also agreed that evidence of potential biomarkers such as sNfL levels, meningeal ectopic lymphoid follicles, and OCT measurements is promising (31–33), and that these biomarkers, together will digital devices, will prove useful in detecting disease progression in the near future.

One limitation of the present consensus statements could be that only experts from the Spanish clinical practice participated in the study. However, consensus statements on the identification of progression by clinical and radiological features and by biomarkers are expected to be a useful resource for neurologist worldwide, who still face the challenge of identifying conversion to SPMS with limited guide and no standard criteria.

## Conclusion

These consensus statements could help clinicians on the early identification of SPMS, in a context where no standard diagnostic criteria are available. Early identification of progression in MS is fundamental since it facilitates a better therapeutic management of the disease. Although by the consensus has been agreed that diagnosis of SPMS should be confirmed based only on clinical assessments, input from cognitive, PROs, imaging assessments, and systematic review of patients' perceptions of their functional status should also be considered for suspecting progression. As research in MS management continues to evolve and potential biomarkers might be validated in the near future, periodic updates of this document should be performed.

## Data availability statement

The original contributions presented in the study are included in the article/Supplementary material, further inquiries can be directed to the corresponding author.

## Author contributions

JM-L, BC, and CO-G: conceptualization, investigation, methodology, project administration, resources, supervision, validation, visualization, and writing-review and editing. AR-A, SE, GI, CD, JR, MH, CC, JP-G, JA, DU, LC-F, and AG-M: investigation, resources, and validation. All authors contributed to the article and approved the submitted version.

## Funding

This work was supported by Novartis. Meetings, data analysis, and medical writing assistance were funded by Novartis. The funders had no role in study design, data collection and analysis, decision to publish, or preparation of the manuscript.

## Conflict of interest

The authors declare the following potential conflicts of interest regarding the research, authorship, and/or publication of this article: JM-L has received grants and consulting or speaking fees from Almirall, Biogen Idec, Celgene, Genzyme, Merck, Novartis, Roche, and Teva. BC has received research support or personal compensation from any commercial entity (for-profit business) for employment, consulting, serving on a scientific advisory board, speaking, or other activities from Biogen, Sanofi, Roche, Merck, Teva, Novartis, Celgene, and Almirall. AR-A has received personal compensation from any commercial entity (for-profit business) for serving on a scientific advisory board or speaking from Merck, Biogen Idec, Roche, Genzyme, Teva, Mylan, and Celgene. SE has received speaker honoraria and consultant fees from Biogen, Novartis, Sanofi Genzyme, Merck, Almirall, Roche, and Teva. GI has received speaking and/or advisory board honoraria from Bayer, Biogen Idec, Novartis, Sanofi, Merck Serono, Almirall, Roche, Actelion, Celgene, and Teva. CD has received speaking and/or advisory board honoraria from Sanofi, Novartis, AbbVie, and Bial. JR has received speaking honoraria and personal compensation for participating on advisory boards from Biogen Idec, Genzyme, Merck Serono, Novartis, Teva, and Sanofi-Aventis. MH has received research support or personal compensation from any commercial entity (for-profit business) for employment, consulting, serving on a scientific advisory board, speaking, or other activities from Biogen, Novartis, Roche, Merck, Teva, and Genzyme-Sanofi. CC has received personal compensation from any commercial entity (for-profit business) for employment, consulting, serving on a scientific advisory board, speaking, or other activities from Teva, Sanofi-Genzyme, Merck, Novartis, Biogen, and Roche. JP-G is a consultant for Bayer, Biogen Idec, Genzyme, Merck Serono, Novartis, Sanofi-Aventis, Teva, Roche, and Almirall. He has participated as a speaker/moderator in meetings and/or symposia organized by Almirall, Bayer, Biogen Idec, Genzyme, Merck Serono, Novartis, Sanofi-Aventis, Teva, and Roche. He has received grants for research projects from Almirall, Biogen Idec, Novartis, and Sanofi-Genzyme. JA has received honoraria for lecturing, travel expenses for attending meetings, or financial support for research from Biogen Idec, Merck Serono, Genzyme, and Novartis. DU has received honoraria for lecturing, courses, advisory boards, or financial support for research from Biogen, Merck, Novartis, Roche, Sanofi, Teva, Bayer, and Almirall. LC-F has received honoraria for lecturing, travel expenses for attending meetings, or financial support for research from Merck, Bayer, Biogen, Novartis, Sanofi-Genzyme, Almirall, Roche, Celgene, Biopas, Ipsen, and Teva. AG-M has received compensation for lecturing, scientific advisory board and consulting from Novartis, Merck, Roche, Emerald, Biogen and Sanofi, and research support from Teva. CO-G has received honoraria for speaking and/or consultancy from Biogen, Sanofi-Genzyme, Merck, Roche, Teva, and Novartis.

## Publisher's note

All claims expressed in this article are solely those of the authors and do not necessarily represent those of their affiliated organizations, or those of the publisher, the editors and the reviewers. Any product that may be evaluated in this article, or claim that may be made by its manufacturer, is not guaranteed or endorsed by the publisher.

## Supplementary material

Supplementary information file – Includes all the supporting files for this submission.
