# Supplementary material for: Consensus on early detection of disease progression in patients with multiple sclerosis
Source: Front Neurol. 2022 Jul 28;13:931014. doi: 10.3389/fneur.2022.931014 (PMC9366521; doi:10.3389/fneur.2022.931014)
Supplement: Supplementary file 1 [file Table_1.docx]

Supplementary Material

## Table S1. Identification of progression by EDSS and functional assessments: consensus in favour

| **Statement** | | **Consensus in favour** | | | |  |
| --- | --- | --- | --- | --- | --- | --- |
|  | | **Degree of agreement** | | **Degree of recommendation** | |  |
|  | | **(%)^1^** | **Median** | **(%)^2^** | **Median** | |
| In patients with clinical and radiological stability receiving immunomodulatory treatment, clinical monitoring: | |  |  |  |  | |
|  | 2. is recommended every 6 months. | 93 | Totally agree | 87 | Recommended | |
|  | 3. will be determined by the clinician on a case-by-case basis. | 80 | Agree | 67 | Recommended | |
| In patients with clinical and radiological stability receiving immunosuppressant treatment, clinical monitoring: | |  |  |  |  | |
|  | 5. is recommended every 6 months. | 73 | Agree | 73 | Recommended | |
|  | 6. will be determined by the clinician on a case-by-case basis. | 93 | Totally agree | 67 | Recommended | |
| In patients with clinical and radiological instability related to the DMT, clinical monitoring: | |  |  |  |  | |
|  | 7. is recommended every 3 months. | 80 | Agree | 73 | Recommended | |
|  | 9. will be determined by the clinician on a case-by-case basis. | 93 | Totally agree | 87 | Recommended | |
| 10. If progression is suspected, the patient should be evaluated every 3 months. | | 80 | Agree | NC | NC | |
| 12. EDSS score is the best measure available in clinical practice to define progression. | | 93 | Agree | 87 | Recommended | |
| 13. Considering the EDSS, a suitable definition of progression would be: An increase in EDSS by 1 point if the baseline EDSS was ≤ 5.5 or by 0.5 points if the baseline EDSS score was ≥ 6. Consider a minimum EDSS of 4, a pyramidal function system of ⩾2 and confirmation of progression for a minimum of 3 months. | | 100 | Agree | 87 | Recommended | |
| 15. Regardless of the variable used, the minimum time to establish the diagnosis of confirmed progression of disability not associated with relapses is 6 months. | | 87 | Totally agree | 80 | Recommended | |
| 17. A confirmed worsening by 2 points in any functional system (except the visual system), even without changes in EDSS, allows to suspect a diagnosis of progression: | | 80 | Agree | 87 | Recommended | |
|  | 19. with a disease duration <10 years. | 93 | Agree | NC | Recommended | |
|  | 21. with a disease duration between 10 and 20 years. | 87 | Agree | 73 | Recommended | |
|  | 23. with a disease duration > 20 years. | 73 | Agree | 80 | Recommended | |
|  | 25. if the patient is <35 years old. | 87 | Agree | 73 | Recommended | |
|  | 27. if the patient is between 35 and 45 years old. | 87 | Agree | 80 | Recommended | |
|  | 29. if the patient is > 45 years old. | 87 | Agree | 80 | Recommended | |
| Diagnosis of progression should be suspected when there is a confirmed 20% time increase in: | |  |  |  |  | |
|  | 31. the 25FTW. | 93 | Agree | 80 | Recommended | |
|  | 33. the 9HPT. | 87 | Agree | 67 | Recommended | |
|  | 35. the 25FTW and the 9HPT. | 100 | Agree | 87 | Recommended | |
|  | 39. the 2MWT. | 87 | Agree | 80 | Recommended | |
|  | 37. the 25FTW and the 9HPT, together with an increase in EDSS. | 100 | Totally agree | 80 | Recommended | |
| 38. Diagnosis of progression can be confirmed when there is a confirmed 20% time increase in the 25FTW and the 9HPT, together with an increase in EDSS. | | 87 | Agree | 74 | Recommended | |
| 41. In a patient capable of walking 500 meters or more without help or rest, a confirmed reduction from 500 to 300 meters should indicate that more accurate progression diagnostic tools should be used. | | 100 | Totally agree | 93 | Essential | |
| 44. Transition from walking independently to needing any kind of support or help to walk should indicate that more precise progression diagnosis tools need to be used. | | 100 | Totally agree | 100 | Essential | |
| 45. If a patient experiences repeated falls, even if the EDSS or other scales remain unchanged, progression of disability should be suspected. | | 100 | Agree | 73 | Recommended | |

*Notes:*

**^1^**Sum of the percentages of responses obtained for "Totally agree" and "Agree". If no consensus on the degree of recommendations was reached (i.e. <66.6%) NC is shown.

**^2^**Sum of the percentages of responses obtained for "Recommended" and "Essential". If no consensus on the degree of recommendations was reached (i.e. <66.6%) NC is shown.

## Table S2. Identification of progression by EDSS and functional assessments: consensus against

| **Statement** | | **Consensus against** | | | |  |
| --- | --- | --- | --- | --- | --- | --- |
|  | | **Degree of agreement** | | **Degree of recommendation** | |  |
|  | | **(%)^1^** | **Median** | **(%)^2^** | **Median** | |
| 1. In patients with clinical and radiological stability receiving immunomodulatory treatment, clinical monitoring is recommended every 3 months. | | 80 | Disagree | NC | Depending on availability | |
| 4. In patients with clinical and radiological stability receiving immunosuppressant treatment, clinical monitoring is recommended every 3 months. | | 67 | Disagree | NC | According to clinical criteria/optional | |
| 8. In patients with clinical and radiological instability related to the DMT, clinical monitoring is recommended every 6 months. | | 67 | Disagree | NC | According to clinical criteria/optional | |
| 16. Regardless of the variable used, the minimum time to establish the diagnosis of confirmed progression of disability not associated with relapses is 12 months. | | 73 | Disagree | NC | According to clinical criteria/optional | |
| 18. A confirmed worsening by 2 points in any functional system (except the visual system), without changes in EDSS, is sufficient to confirm the diagnosis of progression. | | 67 | Disagree | NC | According to clinical criteria/optional | |
| 20. A confirmed worsening by 2 points in any functional system (except the visual system), without changes in EDSS, with a disease duration <10 years is sufficient to confirm the diagnosis of progression. | | 67 | Disagree | NC | According to clinical criteria/optional | |
| Diagnosis of progression can be confirmed when there is a confirmed 20% time increase in: | |  |  |  |  | |
|  | 32. the 25FTW. | 93 | Disagree | 93 | Not recommended | |
|  | 34. the 9HPT. | 100 | Disagree | NC | Not recommended | |
|  | 40. the 2MWT | 93 | Disagree | 66 | Not recommended | |
| 46. If a patient experiences repeated falls, even if the EDSS or other scales remain unchanged, it can be considered as a sufficient criterion for the diagnosis of progression. | | 93 | Disagree | NC | Not recommended | |

*Notes:*

**^1^**Sum of the percentages of responses obtained for "Totally disagree" and " Disagree". If no consensus on the degree of recommendations was reached (i.e. <66.6%) NC is shown.

**^2^**Sum of the percentages of responses obtained for "Not recommended" and " Depending on availability". If no consensus on the degree of recommendations was reached (i.e. <66.6%) NC is shown.

*applying the time each clinician uses to establish confirmed progression.

## Table S3. Identification of progression by cognitive assessments: consensus in favour

| **Statement** | **Consensus in favour** | | | |
| --- | --- | --- | --- | --- |
|  | **Degree of agreement** | | **Degree of recommendation** | |
|  | **(%)^1^** | **Median** | **(%)^2^** | **Median** |
| 1. Since the diagnosis of the disease, a patient must have at least one annual cognitive assessment. | 80 | Totally agree | 67 | Recommended |
| 2. The annual cognitive assessment must include the largest number of domains, so it is recommended to apply at least one intermediate duration battery such as BRB-N. | 93 | Agree | NC | According to clinical criteria/optional |
| 3. If it is not possible to apply an intermediate duration battery annually, a short battery such as BICAMS should be applied. | 73 | Agree | NC | According to clinical criteria/optional |
| 4. If it is not possible to apply a short duration battery annually, a test such as the SDMT should be applied. | 100 | Totally agree | NC | Recommended |
| 6. The increase in the frequency of the cognitive assessment may vary depending on the clinical situation of the patient and as considered by the neurologist. | 100 | Totally agree | 87 | Essential |
| 8. If a neuropsychologist is not available, given the importance of cognitive assessment using a battery (such as BICAMS), the assessment should be performed by another qualified healthcare professional. | 100 | Agree | 87 | Recommended |
| 9. If, after applying a short or intermediate battery, progression of cognitive decline is suspected, a comprehensive neuropsychological study by a neuropsychologist is recommended. | 100 | Agree | 86 | Recommended |
| 10. A confirmed 20% reduction in the SDMT allows to suspect a diagnosis of progression*. | 93 | Agree | 67 | Recommended |
| 12. A confirmed 20% worsening in at least two subtests of the BRB-N or BICAMS battery, having been able to exclude other factors, allows suspecting a diagnosis of progression*. | 87 | Agree | 80 | Recommended |
| 14. A confirmed 20% worsening in time in at least two subtests of the BRB-N or BICAMS battery allows suspecting a diagnosis of progression*. | 87 | Agree | 80 | Recommended |
| 16. An isolated worsening of cognitive function allows suspecting a diagnosis of progression. | 87 | Agree | 67 | Recommended |
| 17. An isolated worsening of cognitive function is not enough to diagnose progression; it is also necessary that other functional systems worsen. | 67 | Agree | NC | Recommended |

*Notes:*

**^1^**sum of the percentages of responses obtained for “Totally agree” and “Agree”. If no consensus on the degree of recommendations was reached (i.e. <66.6%) NC is presented.

**^2^**sum of the percentages of responses obtained for “Recommended” and “Essential”. If no consensus on the degree of recommendations was reached (i.e. <66.6%) NC is presented.

*applying the time each clinician uses to establish confirmed progression.

## Table S4. Identification of progression by cognitive assessments: consensus against

| **Statement** | **Consensus against** | | | |  |
| --- | --- | --- | --- | --- | --- |
|  | **Degree of agreement** | | **Degree of recommendation** | |  |
|  | **(%)^1^** | **Median** | **(%)^2^** | **Median** | |
| 7. In order to obtain a reliable cognitive assessment using a battery, such as BICAMS, it needs to be applied by a neuropsychologist. | 80 | Disagree | NC | According to clinical criteria/optional |  |
| 11. A confirmed 20% reduction in the SDMT is sufficient for the diagnosis of progression*. | 93 | Disagree | 67 | Not recommended |  |
| 13. A confirmed 20% worsening in at least two subtests of the BRB-N or BICAMS battery, after excluding other factors, is sufficient for the diagnosis of progression*. | 80 | Disagree | NC | Not recommended |  |
| 15. A confirmed 20% worsening in time in at least two subtests of the BRB-N or BICAMS battery, after excluding other factors, is sufficient for the diagnosis of progression*. | 87 | Disagree | NC | Not recommended |  |

*Notes:*

**^1^**Sum of the percentages of responses obtained for "Totally disagree" and " Disagree". If no consensus on the degree of recommendations was reached (i.e. <66.6%) NC is shown.

**^2^**Sum of the percentages of responses obtained for "Not recommended" and " Depending on availability". If no consensus on the degree of recommendations was reached (i.e. <66.6%) NC is shown.

*applying the time each clinician uses to establish confirmed progression.

## Table S5. Identification of progression by additional assessments: consensus in favour

| **Statement** | | **Consensus in favour** | | | |  |  |
| --- | --- | --- | --- | --- | --- | --- | --- |
|  | | **Degree of agreement** | | **Degree of recommendation** | |  |  |
|  | | **(%)^1^** | **Median** | **(%)^2^** | **Median** | |  |
| Since the diagnosis of the disease, patients must complete, at least once per year, a scale/questionnaire that assesses: | |  |  |  |  | | |
|  | 1. fatigue. | 73 | Agree | 67 | Recommended | | |
|  | 2. QoL. | 80 | Agree | 67 | Recommended | | |
|  | 3. depression. | 80 | Agree | 67 | Recommended | | |
|  | 4. depression and fatigue. | 74 | Agree | NC | Recommended | | |
| 5. Since the diagnosis of the disease, in case of alterations in the pyramidal functional system, patients must complete a scale that assesses spasticity, at least once per year. | | 74 | Agree | 67 | Recommended | | |
| 6. Changes in fatigue and depression scales unlikely determine the diagnosis of progression. | | 74 | Agree | NC | Recommended | | |
| 7. Changes in the QoL questionnaires should indicate that more accurate diagnostic tools for progression should be used. | | 80 | Agree | 73 | Recommended | | |
| 8. A worsening of spasticity should indicate that more accurate diagnostic tools for progression should be used. | | 87 | Agree | 73 | Recommended | | |
| 9. The patient should be asked proactively and in a structured manner if she/he is experiencing any deterioration or changes in his symptoms that may be considered or suspect progression. | | 100 | Totally agree | 93 | Recommended | | |

*Notes:*

**^1^**Sum of the percentages of responses obtained for "Totally agree" and "Agree". If no consensus on the degree of recommendations was reached (i.e. <66.6%) NC is shown.

**^2^**Sum of the percentages of responses obtained for "Recommended" and "Essential". If no consensus on the degree of recommendations was reached (i.e. <66.6%) NC is shown.

## Table S6. Identification of progression by radiological characteristics: consensus in favour

| **Statement** | **Consensus in favour** | | | |  |  |
| --- | --- | --- | --- | --- | --- | --- |
|  | **Degree of agreement** | | **Degree of recommendation** | |  |  |
|  | **(%)^1^** | **Median** | **(%)^2^** | **Median** | |  |
| 1. A change in the degree of brain atrophy, that is maintained and/or confirmed over time, should lead to suspicion of disease progression. | 80 | Agree | 71 | Recommended | | |
| 2. A change in the degree of brain atrophy, after excluding other relevant physiological factor, should lead to suspicion of disease progression. | 73 | Agree | 79 | Recommended | | |
| 3. A change in the degree of brain atrophy should indicate that more accurate diagnostic tools of disease progression should be used. | 93 | Totally agree | 93 | Recommended | | |
| 4. A change in the degree of spinal cord atrophy, that is maintained and/or confirmed over time, should lead to suspicion of disease progression. | 100 | Agree | 87 | Recommended | | |
| 5. A change in the degree of spinal cord atrophy should indicate that more accurate diagnostic tools of disease progression should be used. | 93 | Totally agree | 93 | Recommended | | |
| 6. The presence of diffuse hyperintensity in the brain white matter or confluence of lesions should lead to suspicion of progression | 80 | Agree | NC | Recommended | | |
| 7. The presence of meningeal ectopic lymphoid follicles should lead to suspicion of progression | 67 | Agree | NC | Recommended | | |

*Notes:*

**^1^**Sum of the percentages of responses obtained for "Totally agree" and "Agree". If no consensus on the degree of recommendations was reached (i.e. <66.6%) NC is shown.

**^2^**Sum of the percentages of responses obtained for "Recommended" and "Essential". If no consensus on the degree of recommendations was reached (i.e. <66.6%) NC is shown.

## Table S7. Identification of progression by biomarkers: consensus in favour

| **Statement** | **Consensus in favour** | | | |
| --- | --- | --- | --- | --- |
|  | **Degree of agreement** | | **Degree of recommendation** | |
|  | **(%)^1^** | **Median** | **(%)^2^** | **Median** |
| 1. The presence of meningeal ectopic lymphoid follicles could be an important biomarker in the future to detect disease progression. | 73 | Agree | NC | Recommended |
| 2. The presence of serum neurofilament light chain (sNfL) levels will be an important biomarker in the near future to detect disease progression. | 87 | Agree | 80 | Recommended |
| 3. OCT measurements will be an important biomarker in the near future to detect disease progression. | 67 | Agree | NC | According to clinical criteria/optional |
| 4. Digital devices, such as wearables, could become relevant tools in the future for the early identification of disease progression. | 100 | Agree | 73 | Recommended |

*Notes:*

**^1^**Sum of the percentages of responses obtained for "Totally agree" and "Agree". If no consensus on the degree of recommendations was reached (i.e. <66.6%) NC is shown.

**^2^**Sum of the percentages of responses obtained for "Recommended" and "Essential". If no consensus on the degree of recommendations was reached (i.e. <66.6%) NC is shown.

## Table S8. Statements were no consensus (i.e. <66.6%) either in favour or against was reached

| **Statement** | **Degree of agreement** | | | |
| --- | --- | --- | --- | --- |
|  | **(%)^1^** | | | **Median** |
| *Identification of progression by EDSS and functional assessments* |  |  | | |
| 11. If progression is suspected, the patient should be evaluated every 6 months. | | | 60 | Agree |
| 14. Regardless of the variable used, the minimum time to establish the diagnosis of confirmed progression of disability not associated with relapses is 3 months. | | | 53 | Disagree |
| 22. A confirmed worsening by 2 points in any functional system (except the visual system), even without changes in EDSS and with a disease duration between 10 and 20 years is sufficient for confirming the diagnosis of progression. | | | 60 | Disagree |
| 24. A confirmed worsening by 2 points in any functional system (except the visual system), even without changes in EDSS and with a disease duration > 20 years is sufficient for confirming the diagnosis of progression. | | | 53 | Agree |
| 26. A confirmed worsening by 2 points in any functional system (except the visual system), even without changes in EDSS and if the patient is > 35 years old is sufficient for confirming the diagnosis of progression. | | | 60 | Disagree |
| 28. A confirmed worsening by 2 points in any functional system (except the visual system), even without changes in EDSS and if the patient is between 35 and 45 years old is sufficient for confirming the diagnosis of progression. | | | 60 | Disagree |
| 30. A confirmed worsening by 2 points in any functional system (except the visual system), even without changes in EDSS and if the patient is > 45 years old is sufficient for confirming the diagnosis of progression. | | | 60 | Disagree |
| 36. Diagnosis of progression can be confirmed when there is a confirmed 20% time increase in the 25FTW + 9HTP. | | | 66 | Disagree |
| 42. In a patient capable of walking 500 meters or more without help or rest, a confirmed reduction from 500 to 300 meters it can be considered as a sufficient criterion for the diagnosis of progression. | | | 60 | Disagree |
| 43. Transition from walking independently to needing any kind of support or help to walk it can be considered as a sufficient criterion for the diagnosis of progression. | | | 60 | Agree |
| *Identification of progression by cognitive assessments* | | |  |  |
| 5. If it is not possible to apply a short duration battery annually, a test such as the SDMT should be applied. | | | 60 | Agree |

**^1^**Sum of the percentages of responses obtained for "Totally agree" and "Agree" or by "Totally disagree" and " Disagree".
